# Association Between Periodontitis and Ischemic Heart Disease

**DOI:** 10.1002/puh2.70450

**Published:** 2026-11-07

**Authors:** Edmond K. Appiah, Joseph Nimako‐Boateng, Kwame Adjei‐Sefah

**Affiliations:** ^1^ Dornsife School of Public Health Drexel University Philadelphia Pennsylvania USA; ^2^ University of Ghana Dental School Accra Ghana; ^3^ Yale University/Bridgeport Hospital Bridgeport Connecticut USA

**Keywords:** ischemic heart disease, odds ratio, periodontal diseases, periodontal medicine, periodontitis, prevalence

## Abstract

**Background:**

Ischemic heart disease (IHD) is a leading cause of global mortality. This study examined the association between periodontitis severity, graded by clinical attachment loss (CAL), and IHD in a representative sample of US adults.

**Methods:**

We conducted a cross‐sectional analysis of the National Health and Nutrition Examination Survey (NHANES) 2013–2014 cycle, including a full‐mouth periodontal examination, of participants aged ≥30 years with complete periodontal and IHD data (*n* = 2807; weighted *n* ≈ 122,396,066). Periodontal status was classified by maximum interdental CAL, using the severity thresholds of the 2018 classification of periodontal diseases: no‐or‐initial‐attachment loss (CAL ≤2 mm; reference), Stage II periodontitis (CAL 3–4 mm), and Stage III/IV periodontitis (CAL ≥5 mm). IHD was defined as a composite of self‐reported coronary heart disease, angina, or heart attack. Weighted prevalence of IHD was computed for each periodontal group, and survey‐weighted logistic regression estimated odds ratios (ORs) for IHD, adjusting sequentially for demographic characteristics (age, sex, race/ethnicity, and family income), smoking, diabetes, hypertension, and other cardiovascular risk factors.

**Results:**

The weighted prevalence of IHD was 5.71% overall and increased across periodontal groups: 2.45% (no‐or‐initial‐attachment loss), 4.66% (Stage II), and 8.82% (Stage III/IV) (*p* = 0.0004). In unadjusted models, Stage III/IV periodontitis was associated with higher odds of IHD than no‐or‐initial‐attachment‐loss (OR 3.84; 95% confidence interval [CI] 1.33–11.07; *p* = 0.003). After full adjustment, the association was attenuated, but the point estimate remained elevated (OR 1.47; 95% CI 0.40–5.39; *p* = 0.05). Older age, current smoking, diabetes, hypertension, and self‐reported bone loss were independent predictors of IHD.

**Conclusion:**

In this nationally representative study, Stage III/IV periodontitis was associated with a higher prevalence of IHD. Although the association was attenuated after multivariable adjustment, the findings suggest a potential link, possibly mediated by shared risk factors and inflammatory pathways.

## Graphical abstract

Ischemic heart disease prevalence increased with periodontitis severity, from 2.45% among adults with no‐or‐initial‐attachment loss to 8.82% among those with Stage III/IV periodontitis. The association was substantially attenuated after adjustment for demographic and shared cardiovascular risk factors.

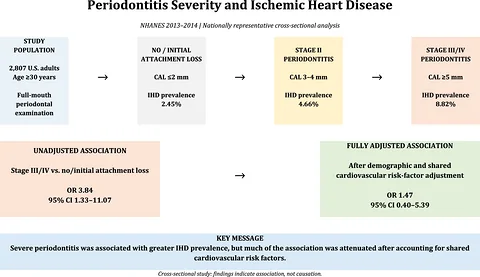

## 1 Introduction

Ischemic heart disease (IHD) remains the leading global cause of mortality and morbidity, with data from the Global Burden of Disease Study 2021 (GBD 2021) reporting approximately 8.99 million deaths in 2021 [1]. Traditional cardiovascular risk factors, including hypertension, diabetes, hyperlipidemia, smoking, and obesity, are widely recognized as major contributors to the development of IHD [2, 3, 4, 5, 6]. However, increasing attention is being given to the potential impact of chronic infection and systemic inflammation as risk pathways in the progression of cardiovascular disease [7].

Periodontal disease refers to inflammatory disorders that involve the supporting structures of the teeth, including the gingiva, periodontal ligament, cementum, and alveolar bone [8]. The condition is caused by polymicrobial dysbiosis, which triggers a persistent and damaging host immune‐inflammatory reaction [9]. Many studies have reported links between poor periodontal health and increased risk of coronary heart disease [10, 11].

The association between periodontitis and IHD has been examined in several previous population‐based studies, including analyses of National Health and Nutrition Examination Survey (NHANES) data. Using NHANES III, Arbes et al. [12] reported that the odds of a self‐reported history of heart attack rose with the extent of periodontal attachment loss, and Hyman et al. [13] showed that much of this association was accounted for by cigarette smoking. More recent analyses of the 2009–2014 cycles, the first to use a full‐mouth periodontal examination, have linked periodontitis defined by the Centers for Disease Control and Prevention/American Academy of Periodontology (CDC/AAP) surveillance case definition [14] to a higher predicted 10‐year atherosclerotic cardiovascular risk [15], and meta‐analyses of cohort studies have reported a modest increase in incident cardiovascular disease among people with periodontitis [16, 17]. These studies, however, have generally contrasted people with and without periodontitis or have relied on composite cardiovascular outcomes or predicted risk scores rather than IHD itself. Fewer have examined IHD across graded severity categories that correspond to the staging thresholds of the 2018 classification of periodontal diseases [18, 19] in the most recent NHANES cycle with a full‐mouth periodontal examination, whereas quantifying through sequential adjustment how much of the crude association is explained by shared risk factors. The present study addresses these gaps.

Given the high prevalence of periodontitis, which affects an estimated 42% of US adults aged 30 years and older [20] and more than one billion people worldwide in its severe form [21], the global burden of IHD, and the possibility of shared inflammatory pathways, clarifying whether periodontitis is an independent risk factor for IHD has important implications. If periodontal status contributes meaningfully beyond traditional risk factors, integrating oral health into cardiovascular prevention strategies might provide additional benefit. This study, using population‐based data, aims to examine whether more severe periodontitis is associated with a higher prevalence of IHD.

## 2 Methods

This study utilized data from the NHANES 2013–2014 cycle. NHANES is a nationally representative, cross‐sectional survey of the noninstitutionalized population in the United States, conducted by the National Center for Health Statistics (NCHS) [22]. The NHANES database is designed to track the health and nutritional status of both adults and children throughout the United States [23]. The survey collects comprehensive health data through interviews, physical examinations, laboratory tests, and dental examinations. The 2013–2014 cycle was selected because it is the most recent NHANES cycle to include a full‐mouth periodontal examination; this protocol, in which probing depth and attachment loss were recorded at six sites on all teeth except third molars, was implemented in the 2009–2010, 2011–2012, and 2013–2014 cycles and was discontinued thereafter [20].

NHANES uses a multistage, stratified probability sampling design, oversampling certain populations. The survey protocol was approved by the NCHS Research Ethics Review Board, and all participants provided written informed consent [24]; the present analysis used publicly available, de‐identified data. The analysis was restricted to adult participants aged 30 years and older who completed both the oral health examination and the medical conditions questionnaire. Participants with missing data on the key exposure (periodontal status) or outcome (IHD) variables were excluded from the analysis.

Periodontal status was assessed clinically during the oral health examination. Eligibility for the NHANES periodontal examination required participants to be at least 30 years old, have a minimum of one natural tooth, and have no medical history requiring antibiotic prophylaxis for periodontal procedures. On the basis of American Dental Association (2008) guidelines, exclusion criteria included a history of heart transplant, artificial heart valve, congenital heart disease (excluding mitral valve prolapse), or bacterial endocarditis. All participants who met the NHANES eligibility criteria and had valid periodontal measurements were retained in the present analysis.

The primary exposure was defined using clinical attachment loss (CAL), a key indicator of periodontal tissue destruction. CAL was recorded in whole millimeters at up to six sites per tooth for all eligible teeth. An array of all interdental (mesial and distal) CAL measurements was used, and the maximum interdental CAL for each participant was determined by identifying the highest valid measurement across all sites. On the basis of the maximum interdental CAL, participants were classified into three mutually exclusive groups using the severity thresholds of the staging framework of the 2018 classification of periodontal diseases and conditions [18, 19]: no‐or‐initial‐attachment loss (maximum CAL ≤2 mm), Stage II periodontitis (moderate; maximum CAL 3–4 mm), and Stage III/IV periodontitis (severe; maximum CAL ≥5 mm).

The reference group comprises participants with clinical periodontal health or gingivitis (no attachment loss) together with those with initial (Stage I) periodontitis (maximum CAL 1–2 mm); these conditions were combined because the NHANES 2013–2014 periodontal examination did not record gingival bleeding, so that health and gingivitis cannot be distinguished [25], and because the primary contrasts of interest were between established (Stage II) and severe (Stage III/IV) periodontitis and the absence of such disease. Classification was based on CAL severity alone; the complexity factors and tooth‐loss criteria of the full 2018 staging framework, and its requirement for attachment loss at two or more nonadjacent teeth, were not applied.

The outcome of interest was a composite measure of IHD, defined as a self‐reported history of any of the following conditions: coronary heart disease, angina/angina pectoris, or heart attack. Participants who reported “Yes” to any of these three conditions were classified as having IHD. Those who reported “No” to all three were classified as not having IHD. Responses of “Refused,” “Don't know,” or missing for all three components resulted in a missing outcome.

Several covariates known to be associated with both periodontitis and IHD were included. These were derived from demographic, questionnaire, examination, and laboratory data. Demographic factors: Age was categorized into three groups (30–49, 50–64, ≥65 years). Sex (male, female) and race/ethnicity (non‐Hispanic White, non‐Hispanic Black, Hispanic, or other) were included. Family income was assessed using the poverty–income ratio (PIR) and categorized as below the poverty line (PIR <1) or at/above the poverty line (PIR ≥1).

### 2.1 Dental Care Utilization

Time since last dental visit was categorized as within 1 year, 1–5 years, or over 5 years/never.

### 2.2 Cardiovascular Risk Factors

Smoking status was derived from the smoking questionnaire and classified as never smoker (fewer than 100 cigarettes in a lifetime), former smoker (at least 100 cigarettes in a lifetime but not smoking at the time of the interview), or current smoker (currently smoking every day or some days). No minimum duration of cessation was required for classification as a former smoker, and smoking intensity and duration were not modeled. Body mass index (BMI) was categorized as underweight (<18.5 kg/m^2^), normal weight (18.5–24.9 kg/m^2^), overweight (25.0–29.9 kg/m^2^), or obese (≥30 kg/m^2^). Self‐reported diabetes status was categorized as normal, prediabetes (borderline), or diabetes. Hypertension was defined by a self‐reported history of being told of high blood pressure. Total cholesterol levels, measured in serum, were categorized as desirable (<200 mg/dL), borderline high (200–239 mg/dL), or high (≥240 mg/dL).

### 2.3 Behavioral Factor

Alcohol use frequency over the past 12 months was categorized as never, occasional (up to weekly), or frequent (more than weekly).

Finally, self‐reported bone loss (derived from the questionnaire item “Ever told you have bone loss around teeth?”) was included as a covariate and was categorized as “Yes” or “No.”

All statistical analyses accounted for the complex survey design of NHANES, incorporating the examination sampling weights, strata, and primary sampling units. Descriptive statistics were used to summarize the characteristics of the study population by periodontal group. Categorical variables were presented as weighted percentages and compared across groups with the Rao–Scott chi‐square test; continuous variables were presented as weighted means with standard errors (SEs). The weighted prevalence of IHD was computed overall and for each periodontal group. To examine the association between periodontitis severity and IHD, we employed survey‐weighted logistic regression models, calculating odds ratios (ORs) and 95% confidence intervals (CIs) with the no‐or‐initial‐attachment‐loss group as the reference. The analysis was conducted using sequential models: A crude model, a model adjusted for demographic factors (age, sex, race/ethnicity, and family income), and a fully adjusted model that included all covariates listed above. Statistical significance was set at a two‐sided *p* < 0.05. All data management and statistical analyses were performed using SAS software (version 9.4; SAS Institute, Cary, NC).

## 3 Results

The analytic sample included 2807 adults, representing approximately 122 million US adults. The mean age was 51.24 years, with older age strongly associated with more severe periodontitis (*p* < 0.0001). Men, non‐Hispanic Black adults, Hispanic adults, and those living below the poverty level had higher rates of advanced disease (*p* < 0.0001). Behavioral factors were significant; participants with infrequent dental visits and current smokers were more likely to have Stage III/IV periodontitis.

Metabolic comorbidities also clustered with severity: Diabetes affected 15.63% of participants with Stage III/IV periodontitis compared with 6.77% of those with no‐or‐initial‐attachment loss (*p* = 0.0008), and hypertension was present in 43.49% versus 24.18% (*p* < 0.0001). BMI and total cholesterol did not differ significantly across groups. The weighted prevalence of IHD was 5.71% overall and rose from 2.45% in participants with no‐or‐initial‐attachment loss to 4.66% in Stage II and 8.82% in Stage III/IV periodontitis (*p* = 0.0004). Further details are presented in Table 1.

**TABLE 1:** Baseline characteristics of the study population by periodontal group (National Health and Nutrition Examination Survey [NHANES] 2013–2014, weighted).

Variable	Overall (*N* = 2807)	No‐or‐initial‐attachment loss (CAL ≤2 mm)	Stage II (CAL 3–4 mm)	Stage III/IV (CAL ≥5 mm)	*p* value
**Age, years, mean (SE)**	51.24 (0.37)	45.2 (0.9)	50.7 (0.4)	56.2 (0.5)	<0.0001
**Age categories, %**					
30–49	48.08	70.42	51.93	33.46	
50–64	33.20	22.55	30.55	41.77	
65+	18.72	7.04	17.52	24.77	
**Male sex, %**	51.85	35.53	55.95	63.97	<0.0001
**Race/Ethnicity, %**					
Hispanic	13.08	7.73	12.00	16.93	
Non‐Hispanic White	71.06	77.92	73.63	63.86	
Non‐Hispanic Black	9.34	7.54	7.68	13.16	
Other	6.52	6.82	6.71	6.06	<0.0001
**Below poverty, %**	16.14	8.06	14.01	22.88	<0.0001
**Last dental visit, %**					
<1 year	66.38	72.56	72.68	52.06	
1–5 years	22.56	22.87	19.99	27.50	
>5 years/never	11.06	4.57	7.32	20.44	<0.0001
**BMI, kg/m** ^2^ **, mean (SE)**	29.48 (0.21)	28.6 (0.3)	29.5 (0.2)	30.0 (0.3)	
**BMI categories, %**					
Underweight	0.91	1.79	0.62	1.21	
Normal	24.70	26.43	25.74	22.11	
Overweight	34.85	31.44	35.93	33.82	
Obese	39.53	40.34	37.71	42.86	0.214
**Smoking status, %**					
Never smoker	55.20	67.30	56.10	43.50	
Former smoker	27.80	22.40	27.90	32.10	
Current smoker	17.00	10.30	16.00	24.40	0.0008
**Diabetes status, %**					
Normal glycemia	86.72	90.45	89.10	80.87	
Prediabetes	2.83	2.79	2.49	3.50	
Diabetes	10.46	6.77	8.41	15.63	0.0008
**Hypertension, %**					
Yes	36.90	24.18	35.60	43.49	<0.0001
**Alcohol use, %**					
Never	15.95	10.37	13.90	21.75	
Occasional (≤weekly)	83.20	89.50	84.98	77.73	
Frequent (>weekly)	0.84	0.13	1.12	0.52	<0.0001
**Bone loss present, %**	12.60	4.54	9.62	20.99	<0.0001
**Total cholesterol, mg/dL, mean (SE)**	194.38 (0.95)	195.8 (1.5)	195.5 (0.9)	191.9 (1.1)	
**Total cholesterol categories, %**					
Desirable (<200 mg/dL)	57.63	59.46	56.65	58.97	
Borderline (200–239 mg/dL)	29.30	29.25	29.90	28.15	
High (≥240 mg/dL)	13.07	11.29	13.45	12.88	0.679
**Ischemic heart disease, %**	5.71	2.45	4.66	8.82	0.0004

*Notes:* Values are survey‐weighted. Means are shown with standard errors (SE); categorical values are within‐group percentages. Periodontal groups are defined by maximum interdental clinical attachment loss (CAL): no‐or‐initial‐attachment loss (periodontal health, gingivitis, or Stage I periodontitis), ≤2 mm; Stage II periodontitis, 3–4 mm; Stage III/IV periodontitis, ≥5 mm. *p* values are from the Rao–Scott chi‐square test (categorical variables) or survey‐weighted linear regression (means).

Abbreviation: BMI, body mass index.

Table 2 shows the unadjusted survey‐weighted logistic regression estimates. The overall effect of the periodontal group on IHD was statistically significant (*p* = 0.0049). Stage III/IV periodontitis was associated with nearly four‐fold higher odds of IHD compared with no‐or‐initial‐attachment loss (OR 3.84, 95% CI 1.33–11.07), whereas Stage II periodontitis was not associated with IHD (OR 1.00, 95% CI 0.71–5.35).

**TABLE 2:** Unadjusted association between periodontal group and ischemic heart disease (National Health and Nutrition Examination Survey [NHANES] 2013–2014, weighted).

Periodontal group	Odds ratio (OR)	95% CI	*p* value
No‐or‐initial‐attachment loss (CAL ≤2 mm)	Reference	—	—
Stage II periodontitis (CAL 3–4 mm)	1.00	0.71–5.35	0.971
Stage III/IV periodontitis (CAL ≥5 mm)	3.84	1.33–11.07	0.003

*Note:* Unadjusted survey‐weighted logistic regression. Overall Wald test for periodontal group, *p* = 0.0049.

Abbreviations: CAL, clinical attachment loss; CI, confidence interval.

In the fully adjusted model (Table 3), the association between Stage III/IV periodontitis and IHD was attenuated and of borderline statistical significance (OR 1.47, 95% CI 0.40–5.39, *p* = 0.05). Older age, current smoking, diabetes, hypertension, and self‐reported bone loss remained independently associated with IHD.

**TABLE 3:** Multivariable association between selected risk factors and ischemic heart disease (National Health and Nutrition Examination Survey [NHANES] 2013–2014, weighted).

Variable	Odds ratio (95% CI)	*p* value
**Periodontal group**		
Stage III/IV periodontitis (CAL ≥5 mm)	1.47 (0.40–5.39)	0.05
Stage II periodontitis (CAL 3–4 mm)	1.17 (0.35–3.93)	0.80
No‐or‐initial‐attachment loss (CAL ≤2 mm)	Reference	—
**Age category**		
30–49 years	0.08 (0.04–0.14)	<0.0001
50–64 years	0.18 (0.11–0.27)	<0.0001
65+ years	Reference	—
**Sex**		
Female	0.51 (0.23–1.14)	0.10
Male	Reference	—
**Race/Ethnicity**		
Hispanic	0.77 (0.49–1.21)	0.25
Non‐Hispanic Black	0.60 (0.38–0.97)	0.036
Other	1.20 (0.59–2.43)	0.61
Non‐Hispanic White	Reference	—
**Family income**		
Below poverty	1.25 (0.75–2.10)	0.39
At/above poverty	Reference	—
**Smoking status**		
Current smoker	2.38 (1.14–4.98)	0.021
Former smoker	0.85 (0.55–1.30)	0.45
Never smoker	Reference	—
**Diabetes status**		
Normal	0.51 (0.29–0.88)	0.016
Prediabetes	0.69 (0.26–1.83)	0.45
Diabetes	Reference	—
**Hypertension**		
No	0.51 (0.36–0.71)	<0.0001
Yes	Reference	—
**Bone loss**		
Yes	1.30 (0.99–1.36)	0.045
No	Reference	—
**Alcohol use**		
Frequent (>weekly)	<0.001 (<0.001 to <0.001)	<0.0001
Occasional (≤weekly)	0.69 (0.42–1.14)	0.15
Never	Reference	—
**Total cholesterol**		
Borderline high (200–239 mg/dL)	1.00 (0.64–1.57)	0.997
High (≥240 mg/dL)	0.48 (0.21–1.10)	0.082
Desirable (<200 mg/dL)	Reference	—

*Note:* Fully adjusted survey‐weighted logistic regression. Time since last dental visit and BMI were included in the model (estimates not shown).

Abbreviations: CAL, clinical attachment loss; CI, confidence interval.

## 4 Discussion

The cross‐sectional analysis of NHANES 2013–2014 data demonstrates a significant association between periodontitis severity and the prevalence of IHD among US adults aged 30 years and older. The prevalence of IHD increased from 2.45% in participants with no‐or‐initial‐attachment loss to 8.82% in those with Stage III/IV periodontitis, with an unadjusted OR of 3.84 (95% CI: 1.33–11.07; *p* = 0.003) for Stage III/IV periodontitis compared with the reference group. After adjusting for demographic factors, smoking, diabetes, hypertension, and other confounders, the association was attenuated but remained borderline significant. These findings indicate that severe periodontitis could play a role in the risk of IHD, possibly mediated by overlapping inflammatory mechanisms and common risk factors [26, 27, 28]. Our results align with prior epidemiological evidence linking periodontitis to IHD. For instance, a meta‐analysis of systematic reviews reported ORs ranging from 1.22 to 4.42 for cardiovascular diseases, including IHD, with a relative risk (RR) of 1.25 for severe periodontitis [16, 17].

The Atherosclerosis Risk in Communities (ARIC) study, which classified periodontal status using the periodontal profile class (PPC) system, a latent‐class taxonomy of seven classes in which the highest class (PPC‐VII) denotes severe tooth loss, found that participants in the severe tooth loss class had a significantly higher risk of incident coronary heart disease, with a fully adjusted hazard ratio of 1.51 [29]. Similarly, Yu et al. [11] found that both incident and prevalent periodontal disease were associated with elevated cardiovascular risks, highlighting the potential long‐term impact of oral inflammation.

The reduction after adjustment highlights the impact of confounders, especially smoking, diabetes, and hypertension, which were significant predictors of IHD in our analysis and are well known to worsen both periodontal and cardiovascular disease [3, 30]. Older age, male sex, lower family income, smoking, and comorbidities like diabetes and hypertension were all strongly associated with severe periodontitis in our baseline characteristics. This confounding effect suggests the necessity to control for these factors to identify the potential independent contribution of periodontitis.

The borderline significant association (*p* = 0.05) after full adjustment, however, suggests that periodontitis may contribute to IHD risk through pathways beyond confounding. The biological mechanisms supporting this association are well‐documented. Periodontal pathogens can induce systemic inflammation through bacteremia, elevating pro‐inflammatory cytokines and C‐reactive protein, which contribute to endothelial dysfunction and atheroma instability [31, 32, 33, 34]. Periodontal pathogens—particularly *Porphyromonas gingivalis*—have been detected in human atherosclerotic plaques, and multiple mechanisms have been identified by which they can directly accelerate atherosclerosis and increase cardiovascular risk [35].

The public health impact is significant, given that periodontitis affects an estimated 42.2% of US adults aged 30 years and older, with 7.8% having severe periodontitis [20], and that heart disease remains the leading cause of death in the United States, accounting for 680,981 deaths (22.0% of all deaths) in 2023 [36] and for the largest share of cardiovascular deaths globally [1, 30]. Periodontal screening in primary care on a regular basis might permit targeted interventions, that is, improved oral hygiene or periodontal treatment, to reduce IHD burden.

The study's strengths are the use of a nationally representative NHANES 2013–2014 dataset with full‐mouth clinical periodontal assessment (CAL), severity categories aligned with the thresholds of the 2018 classification, and sequential covariate adjustment, enabling greater generalizability and reduced selection bias.

However, limitations must be noted. The cross‐sectional design prevents causal inference, and reverse causation or residual confounding may persist. The outcome and several covariates, namely, IHD (coronary heart disease, angina, and heart attack), diabetes, hypertension, bone loss around the teeth, smoking, alcohol use, and time since the last dental visit, were self‐reported and may be subject to recall and misclassification bias. Self‐reported cardiovascular and metabolic diagnoses have, however, shown substantial agreement with medical records: Okura et al. [37] reported kappa coefficients of approximately 0.80 for myocardial infarction, 0.76 for diabetes, and 0.75 for hypertension, and in NHANES III self‐reported hypertension had a sensitivity of 71% and a specificity of 90% against measured blood pressure and medication use [38], whereas in the ARIC study self‐reported diabetes had a sensitivity of 66% and a specificity of greater than 99% against measured glucose and medication use [39].

Self‐reported angina and bone loss around the teeth are less well validated, and misclassification of these variables is likely to have been non‐differential with respect to periodontal status, which would tend to bias associations toward the null. The classification of smoking status did not specify a minimum duration of cessation for former smokers, and smokers were not disaggregated by daily consumption or years of smoking, so residual confounding by smoking cannot be excluded. Periodontal classification was based on the maximum interdental CAL alone, using the severity thresholds rather than the full case definition of the 2018 classification: the complexity factors and tooth‐loss criteria were not applied, the requirement for attachment loss at two or more nonadjacent teeth was not imposed, and the small number of participants with a single natural tooth, who cannot meet that requirement, were retained in accordance with the NHANES eligibility criterion. The reference group combined participants without attachment loss and those with initial (Stage I) periodontitis, because periodontal health and gingivitis could not be distinguished and Stage I could not be separated from health without probing depth and bleeding data; any misclassification arising from these choices would be expected to dilute rather than exaggerate the contrast between the reference group and Stage III/IV periodontitis. Edentulous participants, who may have the most severe history of periodontitis, were not examined and are therefore not represented. The number of IHD cases in the periodontal groups was modest, which resulted in wide CIs for the adjusted estimates. Finally, the data were collected in 2013–2014 and may not reflect current periodontal and cardiovascular health in the United States.

In conclusion, this study found a significant graded relationship between periodontitis severity and the prevalence of IHD in a representative sample of US adults. Although a large portion of this association was explained by shared risk factors, a borderline significant independent association persisted after multivariable adjustment. Longitudinal studies applying the full 2018 case definition of periodontitis are needed to clarify whether periodontitis contributes independently to the development of IHD.

## Author Contributions

**Edmond K. Appiah**: conceptualization, methodology, data curation, investigation, supervision, writing – original draft. **Joseph Nimako‐Boateng**: writing – review and editing. **Kwame Adjei‐Sefah**: writing – review and editing, visualization.

## Funding

The authors have nothing to report.

## Ethics Statement

This study was a secondary analysis of publicly available, de‐identified NHANES 2013–2014 data. The NHANES protocol was approved by the National Center for Health Statistics Research Ethics Review Board, and all participants provided written informed consent. No additional ethics approval was sought for the present secondary analysis because the investigators used only publicly available, de‐identified data and had no direct interaction with participants.

## Conflicts of Interest

The authors declare no conflicts of interest.

## Data Availability

The data used in this study are publicly available from the National Health and Nutrition Examination Survey (NHANES) 2013–2014, administered by the National Center for Health Statistics, Centers for Disease Control, and Prevention. The datasets and accompanying documentation can be accessed through the NHANES website.
